# Complement Factor D as a Strategic Target for Regulating the Alternative Complement Pathway

**DOI:** 10.3389/fimmu.2021.712572

**Published:** 2021-09-09

**Authors:** Jonathan Barratt, Ilene Weitz

**Affiliations:** ^1^Department of Cardiovascular Sciences, University of Leicester, Leicester, United Kingdom; ^2^John Walls Renal Unit, University Hospitals of Leicester National Health Service (NHS) Trust, Leicester, United Kingdom; ^3^Jane Anne Nohl Division of Hematology, University of Southern California Keck School of Medicine, Los Angeles, CA, United States

**Keywords:** complement factor D, factor D, alternative complement pathway, complement activation, inflammation, autoimmune diseases, serine protease inhibitors

## Abstract

The complement system is central to first-line defense against invading pathogens. However, excessive complement activation and/or the loss of complement regulation contributes to the development of autoimmune diseases, systemic inflammation, and thrombosis. One of the three pathways of the complement system, the alternative complement pathway, plays a vital role in amplifying complement activation and pathway signaling. Complement factor D, a serine protease of this pathway that is required for the formation of C3 convertase, is the rate-limiting enzyme. In this review, we discuss the function of factor D within the alternative pathway and its implication in both healthy physiology and disease. Because the alternative pathway has a role in many diseases that are characterized by excessive or poorly mediated complement activation, this pathway is an enticing target for effective therapeutic intervention. Nonetheless, although the underlying disease mechanisms of many of these complement-driven diseases are quite well understood, some of the diseases have limited treatment options or no approved treatments at all. Therefore, in this review we explore factor D as a strategic target for advancing therapeutic control of pathological complement activation.

## Introduction

The immune response, which is orchestrated by complex and interconnected systems and biochemical processes, functions to defend the body against a plethora of disease-causing pathogens and host processes (1). One component involved in the immune response is the complement system, which, although also linked with modulation of adaptive immune responses, plays a vital role in innate immunity. It is especially important in the early stages of life, during which adaptive immunity has not yet been fully developed (2). Despite its evolutionary role in survival and defense against infection (1), the complement system can also be a prominent mediator and/or amplifier of the pathogenesis of many serious diseases, including both inflammatory and autoimmune diseases (3). As such, the complement system has been an important focus for therapeutic intervention (2).

This manuscript reviews one of the components of the complement system: factor D. We emphasize the function that factor D has within both healthy and disease states through its essential role in one of the complement pathways and present important unanswered questions and developments in the field of complement inhibition. Finally, we discuss the rationale for the development of factor D inhibitors and describe how these may help to improve the treatment of complement-driven diseases in the near future.

## The Complement System

**Figure 1:**
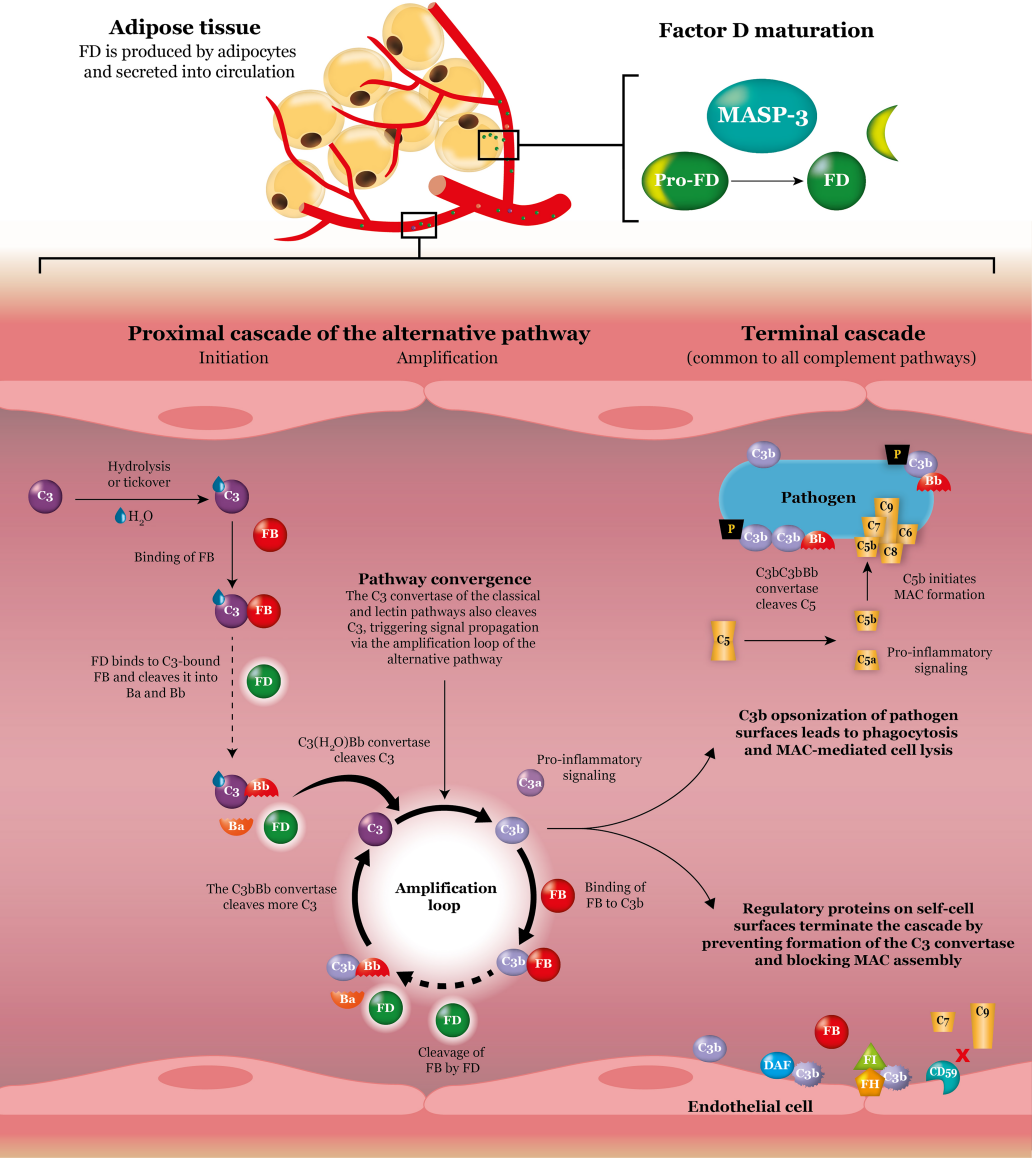
Factor D in the alternative complement pathway. Factor D is produced by adipocytes and secreted into circulation. Once MASP-3 has cleaved off the propeptide and converted factor D into its mature form, factor D is ready to perform its essential function in both the initiation phase and the amplification phase of the alternative complement pathway. First, factor D cleavage of C3-bound factor B is responsible for generation of the C3(H_2_O)Bb convertase, which splits C3 into C3b and the pro-inflammatory signaling anaphylatoxin C3a. Second, factor D cleavage of C3b-bound factor B in the amplification loop is responsible for generation of the predominant C3 convertase C3bBb, which amplifies the signal and creates additional C3b and C3a molecules. The classical and lectin complement pathways converge at the amplification loop of the alternative pathway and as such their signals are also amplified by the action of factor D. Besides incorporation into C3 convertase complexes for signal amplification, C3b also functions to opsonize cells for phagocytosis and, *via* downstream reactions, initiates MAC formation and cell lysis. Inhibition of these activities on self-cell surfaces is controlled by a network of regulatory proteins. Dashed arrows represent the rate-limiting steps. *C, complement; DAF, decay-accelerating factor; FB, factor B; FD, factor D; FH, factor H; FI, factor I; MAC, membrane attack complex; P, properdin*.

The complement system consists of more than 30 cell-associated and plasma-circulating proteins that form three distinct pathways – the classical pathway, the lectin pathway, and the alternative pathway – each with its own activation mechanism (4, 5). The central component of the system, a protein called C3, is cleaved by C3 convertases that are generated by these pathways; C3 is the point at which all three pathways converge (**Figure 1**). The classical pathway is activated by antigen–antibody complexes (4), and the lectin pathway is activated when highly conserved carbohydrate structures present on pathogen surfaces are recognized by pattern recognition molecules in complex with serine proteases (4, 6). In contrast, the alternative pathway, the oldest evolutionary pathway of the system (4), is self-activated by the slow and spontaneous hydrolysis of C3, a process known as *tickover* (7).

Irrespective of the pathway activated, C3 convertases cleave C3 into C3a and C3b in a step that initiates amplification of the signal (5). As the signal is amplified, production of these fragments leads to C3a-mediated inflammation and C3b-mediated opsonization of pathogens for phagocytosis (4). In addition, a cascade of events resulting in cleavage of another complement protein, C5, leads to formation of C5a and C5b. C5b interacts with C6, C7, C8, and C9 to form the membrane attack complex (MAC), which punctures the surface of some pathogens and can result in subsequent cell lysis (4). During amplification of the complement cascade, both C3a and C5a, which are known as anaphylatoxins, are continuously released (3). These proteolytic cleavage fragments, together with other fragments, cause inflammation and induce immune cells to release histamine, interleukin-6, tumor necrosis factor alpha, and other factors that ultimately recruit phagocytes and further activate complement (4, 8).

In addition to the aforementioned actions, which are central to innate immunity, the complement system plays a multi-level role in the adaptive immune response through some of its effector molecules (1) and is important for other physiological processes, such as clearance of host cells after apoptosis (4). Anaphylatoxins produced by the complement system are also responsible for crosstalk with the coagulation pathway. For example, anaphylatoxins such as C5a can contribute to thrombosis by inducing platelet aggregation and tissue factor expression on monocytes (9–11). This in turn leads to the generation of thrombin, a serine protease that is thought to cleave C5 into C5a and C5b, and thereby amplify downstream complement activity and thrombogenicity (12).

Regulation of the complement system is essential for confining complement reactions to pathogenic surfaces when required and preventing the attack of one’s own cells (1, 4). Effective regulation also aids in controlling feedback amplification and the generation of potent effector molecules that may cause collateral tissue damage if produced in excess. Accordingly, host cells and serum are equipped with various proteins that inhibit the complement cascade. For example, proteins such as factor H, factor I, and decay-accelerating factor (DAF), among others, are important for limiting the activity of C3 convertases and preventing their assembly by rapidly inactivating C3b on host cells (1, 3). Moreover, inhibition of MAC assembly by other inhibitors (such as CD59) is also important in situations of rampant complement activation (1, 13).

## The Alternative Complement Pathway

Activation of the alternative pathway is initiated by the spontaneous hydrolysis of C3 (**Figure 1**). Upon self-activation, hydrolyzed C3 (C3(H_2_O)) forms a complex with factor B, thereby enabling factor D to cleave factor B (5). Cleaved factor B in complex with hydrolyzed C3 (C3(H_2_O)Bb) is the initial C3 convertase of the alternative pathway and is responsible for cleaving C3 into C3b and C3a. C3b can then bind to the surface of pathogens that are in close proximity *via* its newly exposed thioester bond (3). Aside from covalently binding, C3b can also interact with surface molecules that recruit C3b.

Once newly generated C3b has bound to the surface of a pathogen, it has three distinct destinies. First, it can act as an opsonin, which, by tagging surfaces as foreign, enhances phagocytosis by host cells (4). Second, it can propagate the complement cascade by entering the amplification loop. In the amplification loop, surface-bound C3b binds to factor B to create the C3bB complex (5). Upon cleavage of this complex by factor D, the predominant C3 convertase of the alternative pathway, C3bBb, is formed and is ready to cleave additional C3 molecules (4). Binding of C3bBb to another protein called properdin (factor P) is known to stabilize the complex by up to about 10-fold (14). The stabilized complex, C3bBbP, can extensively cleave C3 to continue rapidly amplifying the response (5). Third, C3b can bind to C3bBb to form the C5 convertase C3bBb3b. The C5 convertase cleaves C5 into C5a and C5b, which, as discussed previously, ultimately leads to formation of the MAC and causes pathogen lysis (4).

C3b generated by any of the three complement pathways can lead to pathogen opsonization and MAC-mediated cell lysis. However, it is the amplification loop of the alternative pathway that is responsible for propagating the signal and producing large amounts of C3b. In fact, studies have shown that amplification of the alternative pathway is responsible for more than 80% of C5 cleavage when initial activation was *via* the classical pathway (15). As such, the amplification loop is of central importance because it serves to amplify not only the response of the alternative pathway but also the response of the other two complement pathways (5).

### The Role of Factor D Within the Alternative Pathway

As a result of self-activation *via* the spontaneous tickover process, the alternative pathway is permanently active; this low level of activation enables continuous monitoring of the body for invading pathogens (3). Although the pathway is self-activated, factor D plays an essential catalytic role in the formation of C3 convertase and in downstream activation and functioning of the pathway (16). It is not only responsible for catalyzing the first step in the pathway but is also necessary for the propagation of complement activation *via* the amplification loop. Furthermore, because the classical and lectin pathways also use the amplification loop of the alternative pathway, factor D affects the outcomes of these pathways as well.

As factor D is the rate-limiting component of the alternative pathway (17), its concentration ultimately controls the activity and output of this pathway, including that of the amplification loop. This means that alternative pathway–mediated C3a production, opsonization, and cell lysis are all limited by factor D activity.

## Complement Factor D

### Production and Metabolism

Factor D, also known as adipsin, is a 24 kDa serine protease comprising 228 amino acids (18). Unlike most proteins of the complement system, which are synthesized by the liver and immune cells (19), factor D is predominantly produced by and secreted into the bloodstream by adipocytes (18, 20). However, it is also synthesized by macrophages and monocytes (18), as well as by brain astrocytes to a lesser extent (17). The levels of factor D in serum can vary (21), but under normal conditions it is found within the range of 1–2 µg/mL range (16, 22). Under healthy conditions, factor D, like other low molecular weight proteins (23), is filtered through the glomerulus and almost completely reabsorbed within the tubules, where it is then rapidly catabolized intracellularly (24, 25). This is confirmed by the fact that in patients with end-stage kidney disease, plasma levels of factor D increase by about 10-fold because of impaired glomerular filtration (24).

### Maturation and Regulation

Factor D is produced as a proenzyme or zymogen (pro-factor D) that requires subsequent cleavage of a 6–amino acid peptide for maturation (18, 26). Conversion of pro-factor D into its mature form appears to happen rapidly, either during secretion in the secretory pathway or immediately thereafter (27). Although there has been some controversy, maturation of pro-factor D into mature factor D is thought to occur predominantly through the action of activated mannose-binding lectin–associated serine protease-3 (MASP-3), one of the MASPs thought to play a role in the lectin pathway (28, 29). Despite the need for enzyme-mediated maturation, however, factor D predominantly exists in its mature form in resting blood (16, 29), likely because MASP-3 has no physiological inhibitors (28).

With mature factor D being the predominant form in resting blood, and because it has no known endogenous inhibitors itself (30), a high level of control is essential to prevent it from inappropriately cleaving endogenous proteins other than its substrate. As such, mature factor D is locked into an inactive state by a self-inhibitory loop (31–33). This loop dictates the enzyme’s low reactivity and extreme specificity for its substrate, factor B (34). Importantly, factor B can only be cleaved by factor D when factor B is bound to C3b or C3(H_2_O). Upon binding to and cleaving factor B, factor D is not permanently incorporated into the complex but is instead recycled in a reversible reaction (17, 31). Accordingly, the kidney plays an important role in regulating the concentration of factor D *via* glomerular filtration (23).

## Non-Immunological Roles of Factor D

**Figure 2:**
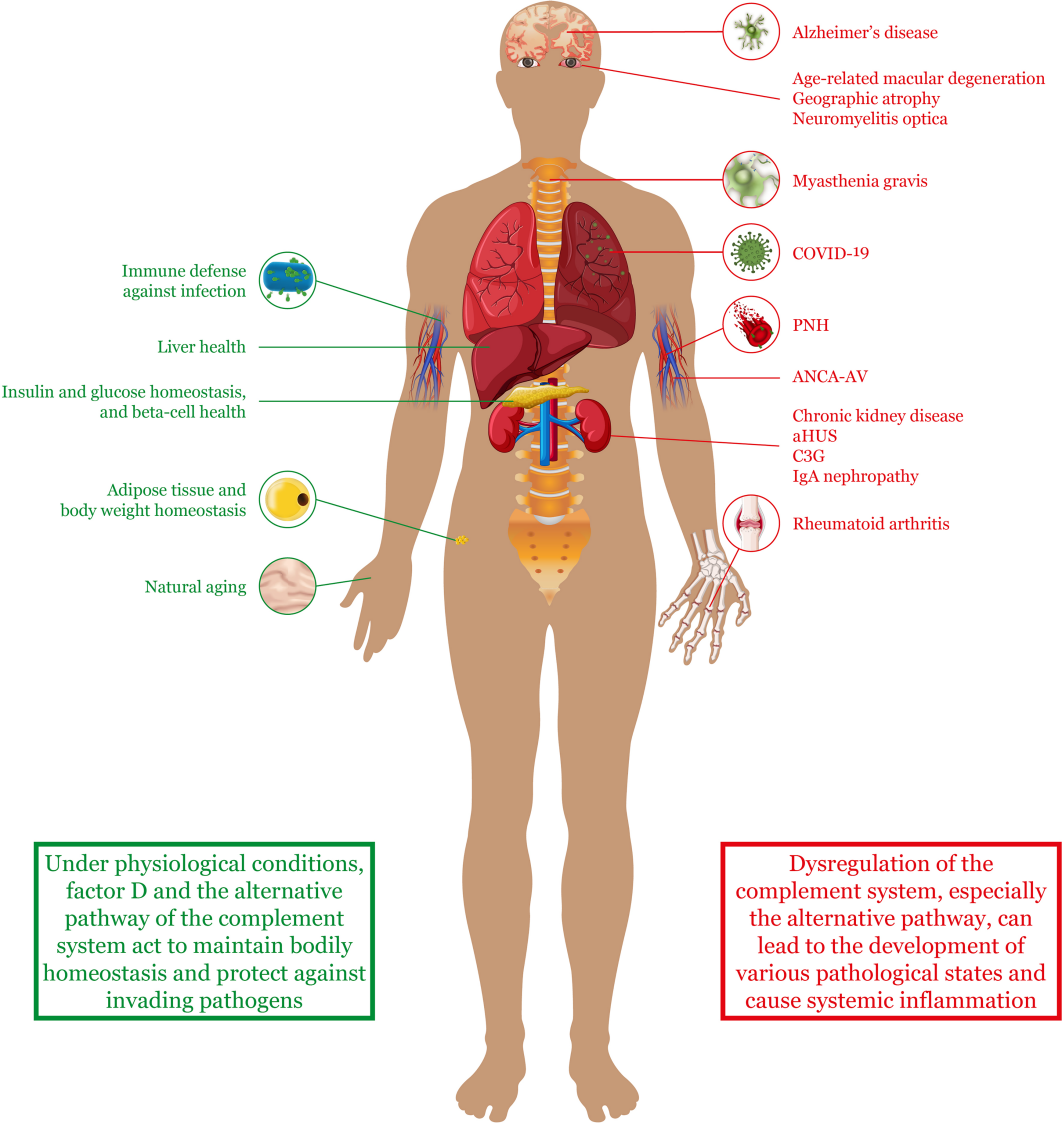
The alternative complement pathway in health and disease. Factor D and the alternative complement pathway have been implicated in both healthy states and disease states. Under normal conditions, the pathway helps to protect against invading pathogens and maintain homeostasis and health of various tissues and organs. However, upon dysregulation, the alternative complement pathway can contribute to the pathogenesis of various diseases throughout the body. Although these diseases can affect specific organs or tissues (as indicated in the figure), many of them are also characterized by systemic complications and widespread inflammation. *aHUS, atypical hemolytic uremic syndrome; ANCA-AV, anti-neutrophil cytoplasmic antibody–associated vasculitis; COVID-19, coronavirus disease 2019; C3G, complement 3 glomerulopathy; PNH, paroxysmal nocturnal hemoglobinuria*.

In addition to host defense against pathogens, the alternative pathway and factor D have been implicated in various other physiological processes (**Figure 2**). For example, C3b-mediated opsonization is known to be responsible for marking damaged liver cells for removal by phagocytes after acute liver injury (35). This process enables a scaffold for newly formed cells to develop and helps to prevent persistent inflammation. The alternative pathway and factor D have been shown to be essential in this process (35, 36). Nonetheless, a fine balance in the level of activation of the alternative pathway is required because an overactive complement cascade is also known to cause extensive hepatic cell death, persistent inflammation, and injury to the liver (36, 37).

More recently, factor D has also been implicated in the aging process of the skin (38). During aging, it is known that the extracellular matrix of the dermal layer deteriorates as the quantity of senescent cells increases (39). Using co-cultured dermal fibroblasts, Ezure et al. showed that factor D gene expression substantially increased in senescent fibroblasts, and that this increase was at least partly responsible for gene expression changes in younger fibroblasts (38). They also showed that factor D gene and protein expression in human skin samples was higher in older subjects than in younger subjects.

Another tissue in which factor D plays a role is adipose tissue. Adipocytes are energy reservoirs that play an important role in energy balance. Not only are they responsible for lipolysis but they are also involved in glucose uptake and triglyceride synthesis (40). Interestingly, factor D is not the only component of the complement system that is produced in adipose tissue. Other factors, including C3 and factor B, are also expressed to some degree in this tissue, where they have been found to activate the proximal part of the alternative pathway (i.e. upstream of C5 cleavage) in the absence of pathogens (19). In fact, factor D has been shown to be important for adipocyte differentiation and lipid accumulation *via* C3a signaling (40). In contrast, no activation of the terminal or lytic part of the pathway is observed, because proteins such as C5 are not expressed in adipose tissue.

As part of their role in energy homeostasis, adipocytes also have endocrine function. In response to certain stimuli, they secrete regulatory molecules that play a role in the metabolic function of other tissues (41). These regulatory molecules include fatty acids and adipokines such as factor D. It has been shown that by controlling the production of C3a *via* the alternative pathway, factor D indirectly induces insulin secretion from pancreatic beta cells when glucose levels are elevated (42). Furthermore, factor D/C3a signaling has been found to preserve islet beta cells by blocking cell dedifferentiation and death (20). It has even been shown that higher levels of circulating factor D are associated with a lower risk of developing diabetes in middle-aged adults.

## Factor D Deficiencies

Deficiencies of the complement system have been described for many of its components, including factor D, factor B, properdin, and factors H and I (21, 22, 43). Complement deficiencies have been associated with increased susceptibility to autoimmune disorders and to recurrent infections, including infections by meningococcal disease–causing pathogens such as *Neisseria meningitidis* (43, 44).

Deficiency in components of the proximal part of the alternative pathway, such as factor D, can lead to an inability to opsonize invading pathogens and to insufficient formation of the MAC. This ultimately limits both phagocytosis and lysis of the invaders. As such, it is not surprising that complete deficiency of factor D has been identified as a risk factor for serious bacterial infections (21). For example, complete factor D deficiency due to a Ser42Stop mutation in both alleles of the gene was observed in a Dutch individual suffering from meningitis. The complete factor D deficiency was linked with a decreased ability to opsonize and phagocytose bacteria and was also observed in other family members. Other case studies have also found factor D deficiencies to be associated with *N. gonorrhoeae* and with various respiratory infections (22, 43). Although the underlying factor D mutations are not always the same, they likely result in an unstable protein or an abnormally folded protein that cannot be secreted (45). Mutations in both alleles are required for complete factor D deficiency, and therefore the mode of inheritance is autosomal recessive (21, 22).

## Dysregulation of Complement Activation in Autoimmune Diseases Where the Alternative Complement Pathway Plays an Important Role

Complement cascade dysregulation can be the pathogenic cause of, or can predispose individuals to, a plethora of chronic human diseases that can affect different organs and systems ranging from the eyes (4) to the kidneys (46) (**Figure 2**). Diseases associated with poorly regulated complement include, but are not limited to, age-related macular degeneration (27), chronic inflammation such as inflammatory arthritis (47), sepsis (48), and lupus nephritis with thrombotic microangiopathy (49). Even small disruptions to the fine balance between activation and regulation of the complement system can lead to uncontrolled inflammation and cell death (4, 50). With the amplification loop of the alternative pathway playing a central role in escalating complement activation, the alternative pathway and factor D contribute to the pathogenesis of many of these diseases.

Complement dysregulation can occur as a result of genetic mutations or the development of autoantibodies to components of the system, both of which can lead to erroneous activation or insufficient control of pathway signaling (4, 51). Therefore, screening for mutations in components of the complement system can be important for diagnosing some complement-driven diseases (3) and may even help to inform treatment decisions. Although complement dysregulation can lead to or contribute to an extremely broad range of diseases that have unique presentations, here we will briefly describe some of the autoimmune disorders that are largely mediated by uncontrolled alternative pathway amplification.

### Paroxysmal Nocturnal Hemoglobinuria

PNH is a rare and life-threatening acquired hemolytic disorder frequently characterized by complement-mediated red blood cell destruction and venous thrombosis (50, 52). Other consequences of this disorder include chronic kidney disease and bone marrow failure (53).

In most patients, PNH is caused by a somatic mutation in the phosphatidylinositol glycan complementation class A (PIG-A) gene within long-lasting hematopoietic stem cells (50, 54). An array of mutations in the PIG-A gene have been described, but usually just a single mutation (54), together with the expansion of the hematopoietic stem cells in which it occurs (53), is responsible for the disorder.

Mutations in the PIG-A gene affect the biosynthesis of glycosylphosphatidylinositol (GPI), a glycerophospholipid that helps to anchor surface proteins to the cell membrane (50). A lack of GPI therefore leads to a deficiency in membrane-anchored surface proteins such as DAF (CD55) and CD59, which act to protect cells from complement attack. Whereas DAF accelerates the decay of C3 and C5 convertases, CD59 prevents assembly of the MAC (53, 55). Therefore, a chronic reduction or absence of these proteins on hematopoietic stem cells leads to uncontrolled complement activation by the alternative pathway on blood cells and platelets (50). This ultimately results in cell lysis and possible thrombosis – the main characteristics of PNH. Although the genetic defect that causes PNH only affects circulating blood cells, lysis of these cells leads to a great deal of downstream complications, including thrombosis, pulmonary hypertension, kidney dysfunction, erectile dysfunction, abdominal and thoracic pain, fatigue, and thrombosis-induced neurological complications (56).

Therapeutic options for PNH include eculizumab and ravulizumab, two humanized monoclonal antibodies that neutralize C5 and impede its conversion into C5a and C5b (50, 57). Treatment with these antibodies leads to a reduced risk of thrombosis and greatly improves outcomes for patients. For example, patients with PNH without bone marrow failure may have a near-normal life expectancy (53). A more recent treatment option for patients with PNH is the C3 inhibitor pegcetacoplan, which was approved by the Food and Drug Administration (FDA) in May 2021 for the treatment of adult patients with PNH, including those switching from anti-C5 therapy (58).

### Complement 3 Glomerulopathy

Complement dysregulation, and most commonly dysregulation of the alternative pathway, is known to cause or accentuate several different inflammatory glomerular diseases (59). In these diseases, glomerular injury leads to the appearance of hematuria and proteinuria and ultimately to the development of progressive chronic kidney disease.

One of these diseases is C3G, a rare, heterogeneous, complement-mediated disease in which dysregulation of the alternative pathway causes poorly controlled complement activation (60). Dysregulation in C3G can be attributed to genetic abnormalities or to the development of autoantibodies. The genetic abnormalities are predominantly acquired and tend to result in fluid-phase dysregulation of the pathway (53). Mutations of C3, factor B, factor H, and factor I have all been found in patients with C3G. Similarly, autoantibodies of varying specificity have been found, but the most frequently occurring autoantibody is known as C3 nephritic factor, which increases the half-life of the C3 convertase.

C3G is generally characterized by deposits of C3b on the glomerular basement membrane; however, factors of the terminal part of the pathway are also present (59). Abnormal serum levels of various complement components have also been found in patients with C3G (61), likely due to the underlying dysregulation of the alternative pathway.

No single treatment has been found to be effective for all patients with C3G, and, in an ideal world, treatment would be tailored to each patient according to the underlying disease mechanism (59). Treatment options include plasma infusion or exchange to replace mutated proteins or remove unwanted autoantibodies; immunosuppressive therapy; and complement-inhibiting antibodies that limit terminal pathway activation. Furthermore, several Phase II clinical trials of additional treatment options for C3G are ongoing; these include investigations of the efficacy and safety of inhibitors of factor D (NCT03459443), factor B (NCT03955445), C3 (NCT04572854), and the C5a receptor (NCT03301467) (62–65).

### Atypical Hemolytic Uremic Syndrome

Hemolytic uremic syndrome is a thrombotic microangiopathy characterized by microangiopathic hemolytic anemia, thrombocytopenia, and microvascular glomerular thrombosis that can result in acute kidney injury (59). The atypical form of the disease, or aHUS, is not self-limiting but rather a systemic disorder that often recurs.

Although aHUS can occur spontaneously with the development of autoantibodies, inherited germline mutations are more commonly the underlying cause (53, 59). In fact, functional deficiencies in various proteins such as factor H, factor I, C3, and factor B (5, 59), as well as thrombomodulin (an endothelial glycoprotein that attenuates activation of the alternative complement pathway within blood vessels) (66), have all been implicated in the pathogenesis of aHUS. Although mutations in thrombomodulin are involved in approximately 5% of all aHUS cases (67), mutations in factor H are the most common predisposing factor (53). Mutations in factor H typically occur within the region that is responsible for mediating cell surface binding and interaction with C3b (59, 68), and therefore they prevent factor H from blocking complement-mediated activation on vascular endothelia and blood cells (59). This is the reason that, unlike in C3G, complement regulation in aHUS tends to be affected more at cell surfaces than in the fluid phase (53). With respect to C3, mutations can either restrict the binding of regulatory factors or lead to a greater affinity for factor B, both of which ultimately cause poorly controlled complement activation on platelets and the glomerular endothelium (53). Importantly, neither genetic mutations or autoantibodies are necessarily a causative factor of aHUS, but rather, together with other genetic factors and environmental precipitants (e.g. infection), they predispose individuals to developing the disease.

Current treatment options for aHUS include plasma exchange to remove autoantibodies and inhibition of C5, the latter of which is more effective and helps to stabilize platelet counts, improve kidney function, and stop hemolysis (59). Although treatment of aHUS with eculizumab blocks MAC formation and the immunological consequences of C5a, the more direct effects of C3 cleavage are maintained (i.e. C3b-mediated opsonization and C3a-mediated pro-inflammatory signaling).

### Geographic Atrophy

GA is an advanced form of age-related macular degeneration in which patients experience irreversible, progressive loss of vision (4). In fact, GA is the most common cause of vision loss in the developed world (69).

Retinal cell death in GA is caused when environmental stressors and oxidative stress trigger inflammation *via* multiple pathways, including the complement and NLRP3 inflammasome pathways (4). In fact, a vast array of evidence supporting a role for complement dysfunction in the pathogenesis of GA has been reported. This includes the discovery of genetic variants of regulator genes such as factor H in genetic studies, the presence of high levels of complement activation products in patients with GA (4), and associations of both single nucleotide polymorphisms and elevated levels of factor D in female patients with the disease (27).

### Anti-Neutrophil Cytoplasmic Autoantibody–Associated Vasculitis

ANCA-AV is a multisystem autoimmune disease that encompasses three distinct conditions, each characterized by inflammatory-cell infiltration into blood vessels and subsequent death of blood vessel cells (70). Patients with ANCA-AV present a broad range of symptoms depending on the specific condition they have, but symptoms can include skin rash, nasal crusting and epistaxis, neuropathy, asthma, peripheral blood eosinophilia, and kidney involvement.

Although the pathogenesis of ANCA-AV is multifactorial, autoantigens produced by neutrophils play an important role in triggering the disease (2, 71). Several autoantigens have been identified at different frequencies in patients with ANCA-AV and include myeloblastin, myeloperoxidase, lysosomal-associated membrane protein 2, and proteinase 3. Upon translocation to the neutrophil cell surface, these autoantigens are recognized by autoantibodies that subsequently lead to complement activation and generation of the anaphylatoxin C5a (70, 72). C5a then recruits further neutrophils to the area in a perpetuating cycle that ultimately results in the death of blood vessel cells.

The role of the alternative complement pathway in ANCA-AV disease pathogenesis and inflammation has been supported by both *in vitro* and *in vivo* data (72, 73). Furthermore, a meta­analysis from 2020 that examined data on the levels of complement proteins in patients with ANCA-AV showed that complement components – including alternative pathway–specific components such as factor B – were increased in patients with active ANCA-AV when compared with control participants (74).

Treatment of ANCA-AV has typically focused on inducing and maintaining remission of the disease with the use of glucocorticoids and cyclophosphamide or rituximab (70). However, more recently efforts have been made to target the complement system and the C5a axis specifically (75). For example, the safety and efficacy of the C5a inhibitor avacopan versus prednisone was evaluated in a Phase III clinical trial of patients with ANCA-AV in which all patients received cyclophosphamide or rituximab. Despite avacopan not being superior to prednisone in terms of disease remission at Week 26, it was found to be superior in terms of sustained remission at Week 52 (76).

## Therapeutic Targeting of the Complement System

As an essential player in both the innate and the adaptive immune response, the complement system is an attractive therapeutic target for the treatment of autoimmune and complement-driven diseases. In fact, inhibition of this system can reduce systemic inflammation and impair immune attack against one’s own cells and tissues (77). Furthermore, the complement system is often an early contributor to the pathogenesis of many diseases and, as such, is a sensible target for intervention (2). Although the complement system offers many possible targets, serine proteases and anaphylatoxin receptors have been hypothesized to be highly conducive to drug targeting (2, 50).

The design of therapeutic candidates is challenging. Treatments need to not only be highly selective and potent but also be associated with minimal adverse effects and have low production costs (50). Despite these challenges, various therapeutic agents covering multiple targets within the complement system are being investigated for the treatment of diseases that are associated with complement dysregulation (2). These include serine protease inhibitors, soluble complement regulators, therapeutic antibodies, small functional complement inhibitors, and anaphylatoxin receptor antagonists. Soluble complement regulators, which aim to suppress complement activation by replacing or supplementing endogenous regulatory proteins, can be purified from plasma or produced recombinantly but are not a very effective treatment option for patients with autoantibodies against the supplemented regulatory protein (77). Another therapeutic option is the use of small interfering RNA that directly suppresses production of the target protein. One example is the antisense factor B inhibitor IONIS-FB-LRx, which is currently being examined in a Phase II clinical trial (NCT04014335) for the treatment of adult patients with IgA nephropathy (78), another disease in which complement activation – principally *via* the alternative and lectin pathways – plays a critical role in disease pathogenesis (79). Although potentially beneficial for patients requiring chronic inhibition, this type of treatment is more difficult to reverse. Importantly, each of these approaches also comes with its own mechanistic advantages. For example, blocking the action of anaphylatoxins using anaphylatoxin receptor antagonists enables excessive inflammatory responses to be controlled without losing the ability to opsonize and destroy pathogens (50).

Therapeutic antibodies targeting the complement system have also been studied extensively in clinical trials, with some achieving market approval. For example, eculizumab is approved by both the FDA and the European Medicines Agency (EMA) for the treatment of aHUS and PNH and has revolutionized the treatment of patients with these diseases (80). It is also approved by both agencies for the treatment of adult patients with some forms of myasthenia gravis and neuromyelitis optica (81, 82). More recently, ravulizumab, a second-generation intravenous C5 monoclonal antibody requiring less frequent administration than eculizumab, has also been licensed by the FDA and the EMA, thereby offering patients a less burdensome alternative (80).

As previously mentioned, the cyclic peptide inhibitor of C3, pegcetacoplan, was approved by the FDA in May 2021 for the treatment of adult patients with PNH (58), thereby further expanding the list of approved treatment options that target the complement system. Despite these advances, however, there are still many unmet needs in the realm of complement-driven diseases. This is exemplified by the fact that for some of these diseases, no approved or effective treatments are currently available (4, 83). To fulfill these unmet needs, many other treatments targeting the complement system are under development, including factor D inhibitors that specifically aim to block alternative pathway–mediated complement amplification.

### Limitations of Current Therapies, Targets, and Approaches

When developing novel treatments for complement-driven diseases, it is important to consider which component of the system may be the most appropriate target. For example, although inhibition of C5 impedes formation of the MAC, this inhibition does not block the pro-inflammatory and opsonization actions of C3, because C5 acts downstream of C3 as part of the terminal cascade (4). Therefore, anti-C5 therapy may have limited effects in diseases where the involvement of C3 in the pathogenesis is high.

In PNH, for example, although eculizumab is the current standard of care, response to the drug is quite variable, and ongoing proximal complement activation still occurs (55). In fact, about two-thirds of patients with PNH treated with eculizumab still experience some level of anemia and may even require red blood cell transfusions. Anemia in these patients can be the result of immune-mediated bone marrow dysfunction, residual or breakthrough intravascular hemolysis, and/or the development of C3-mediated extravascular hemolysis. Importantly, C3-mediated extravascular hemolysis is in part mechanistically associated with anti-C5 treatment and occurs in about 25%–50% of patients. It is the result of persistent and uncontrolled activation of proximal complement, which leads to the opsonization of red blood cells by C3b and other C3-derived fragments and the subsequent removal of these cells in the liver or spleen by phagocytosis.

Limitations of C5 inhibition in the treatment of PNH have recently been highlighted in the Phase III clinical trial (NCT03500549) in which pegcetacoplan and eculizumab were compared (84). In this study, pegcetacoplan showed superiority to eculizumab in improving both hemoglobin levels (3.84 g/dL adjusted mean difference; *P*<0.001) and clinical and hematologic outcomes. Although pegcetacoplan was shown to provide control of both intra- and extravascular hemolysis, protection against extravascular hemolysis may have been the result of this upstream inhibitor providing more comprehensive protection. Nonetheless, it is also important to note that pegcetacoplan is a broad complement inhibitor and does not specifically target any one of the individual pathways.

As a monoclonal antibody, eculizumab also has high production costs, limiting both its access and its off-label use for other diseases (77, 80). Antibodies are also large molecules that do not allow for oral administration. In fact, the biweekly requirement for long intravenous infusions with eculizumab poses a considerable treatment burden for patients (2). Although ravulizumab does have lower yearly costs and a reduced administration frequency compared with eculizumab, it still requires intravenous infusions every eight weeks (80). Pegcetacoplan, on the other hand, is priced similarly to ravulizumab (85) but has the advantage of being administered subcutaneously (58).

These are not the only limitations for complement-targeted treatments. For example, in limited cases, treatment of patients with PNH or aHUS using eculizumab is not effective because of a rare polymorphism in C5 that prevents antibody binding (53, 86). Furthermore, a lack of target specificity can also be a problem in some cases. Nonetheless, it is important to note that antibodies are generally specific and have limited off-target effects (77).

These limitations, together with the fact that dysregulated complement can lead to a multitude of diseases with highly differing symptoms and consequences, highlight the necessity for alternative treatment options that target other components of this complex system.

## Factor D as a Strategic Target in Complement-Driven Diseases

Factor D has emerged as an attractive target for effective and specific pharmacological inhibition of the alternative pathway. It is not only the rate-limiting component of this pathway but is also required for one of the initial steps (25). In addition, factor D is the smallest complement protein, has the lowest concentration, and has a highly selective function with only one natural substrate. In contrast, C3 is one of the most abundant proteins in plasma, accounting for about 2% of all proteins (61). Altogether, these features highlight the potential that factor D has as a target for the treatment of diseases mediated by the alternative pathway.

As factor D is an upstream target in the proximal part of the alternative pathway, its inhibition would impede alternative pathway–mediated cleavage of C3 into C3b and C3a. This could have a more profound effect than inhibiting the pathway further downstream, at C5 for instance, because downstream inhibition would not block C3b-mediated opsonization or C3a-mediated inflammation (77). Importantly, although inhibiting factor D would also block signal amplification of the classical and lectin pathways, cleavage of C3 by the C3 convertase specific to these two pathways would not be blocked. Similar effects may also be expected upon inhibition of factor B because it too is specific to the alternative pathway. In fact, one small-molecule inhibitor of factor B called iptacopan (LNP023) was shown to prevent complement activation in sera from C3G patients and hemolysis of human PNH erythrocytes *in vitro* (87), and is currently under investigation in clinical trials for the treatment of various autoimmune diseases including C3G, IgA nephropathy, aHUS, and PNH (87, 88). It will be interesting to see how inhibitors of these alternative pathway–specific factors actually compare in the clinical trial setting. Nonetheless, advantages of blocking the complement system upstream of C5 have been shown in clinical trials of patients with PNH (80, 84). In these studies, recently approved pegcetacoplan was shown to control both intra- and extravascular hemolysis.

As mentioned, inhibition of factor D would selectively target the alternative pathway but would not affect the initiation or terminal functioning of the lectin and classical pathways. This is important because defense against invading pathogens is partly dependent on antibody-mediated activation of the classical pathway and terminal pathway–mediated bacterial lysis, both of which would be maintained, at least to some extent, with factor D inhibition (89). In this way, factor D inhibition could potentially be associated with a reduced risk of infections compared with C5 blockade (90) or even C3 blockade. In fact, infections have been reported in eculizumab-treated patients with PNH and aHUS who have been vaccinated against meningococcus. Infection can further complicate the clinical picture by inducing complement activity and inflammation, even in patients who otherwise have their disease under control (77). *In vitro* assays using whole blood showed that factor D inhibition has less of an impact on bacterial clearance compared with C5 inhibition, partly because the anaphylatoxin C5a is required for activating leukocytes to phagocytose pathogens (91). Case studies of patients with deficiencies in factor D have also indicated that partial deficiency is unlikely to increase susceptibility to infections (21).

Moreover, high plasma levels of the factor D protein itself have also been implicated in the pathogenesis of some cases of complement-mediated diseases, such as age-related macular degeneration (27). In these cases, inhibiting factor D may help to control disease symptoms and severity irrespective of whether disease pathology is initiated downstream in the pathway.

### Potential Limitations of Targeting Factor D

One possible drawback of factor D inhibition is that although it would block signaling through the alternative pathway and limit amplification of the lectin and classical pathways, it would not fully block all complement activation (55). The same would be true upon inhibition of factor B, and although this may be positive for ensuring some level of activity against invading pathogens as discussed previously, it may also limit treatment effectiveness at attenuating disease symptoms. For example, although dysregulated alternative pathway amplification is the principal pathogenic mechanism in PNH, activation of the other complement pathways upon infection could also contribute to complement activation and hemolysis.

It has also been suggested that the requirement for factor D under certain physiological conditions could be bypassed if another plasma protease cleaves and activates C3 (55). Indeed, other factors such as the contact immune surveillance protein kallikrein are capable of activating C3 (92). As such, these proteins could render factor D inhibition ineffective at limiting complement dysregulation under certain conditions.

The self-inhibitory loop of factor D could also pose a challenge by potentially limiting access of small-molecule inhibitors to the catalytic site. Furthermore, the rapid turnover of factor D may mean that high levels of an inhibitor are required for target saturation. However, the latter limitation would also apply to other complement components with a high turnover, including C3 (93).

Finally, Wu et al. showed that even very low levels of factor D in serum are sufficient for activity of the alternative pathway (94). Therefore, achieving complete inhibition of factor D may be essential for the treatment of complement-mediated diseases.

### Factor D Inhibitors

Inhibitors of factor D are classified as proximal complement inhibitors because they interfere with the early phases of complement activation. Various efforts have already been made to develop and clinically investigate factor D inhibitors (51, 95). Although some of these efforts have involved taking advantage of structural information to develop highly specific small-molecule inhibitors (95, 96), others have evaluated the feasibility of using anti–factor D antibody fragments (97).

Small-molecule inhibitors have the clear advantage of an oral administration route (55) and, unlike antibodies, have the potential to achieve adequate and sustained systemic inhibition without disrupting steady-state levels of the target protein (90). As they are cleared from the system rapidly, small-molecule inhibitors also permit rapid restoration of normal levels if required (e.g. during infection) (89). They also offer more flexible regimens for dosing compared with antibodies because doses can be adjusted more easily. This could be beneficial to patients who are predisposed to developing a disease, because a low prophylactic dose may help to prevent disease onset and progression (90).

With structure-based design and biochemical assays, extensive work has led to the development of various reversible, small-molecule inhibitors of factor D (51, 90), many of which have undergone further optimization for improved pharmacokinetic parameters, potency, and selectivity (51). However, only a limited number of small-molecule factor D inhibitors have progressed into clinical trials for the treatment of alternative pathway–mediated diseases (**Table 1**).

**Table 1:** Small-molecule factor D inhibitors in clinical trials.

Compound name	Company name	NCT number	Phase of development	Indication	NCT reference
**ALXN2040** (ACH-4471/danicopan/ACH-0144471)	Alexion Pharmaceuticals	NCT04469465	Phase III	PNH	(98)
		NCT03472885	Phase II		(99)
		NCT03053102			(100)
		NCT03181633			(101)
		NCT03369236	Phase II	C3G (C3GN + DDD)	(102)
		NCT03459443		C3G (C3GN + DDD) and IC-MPGN	(62)
		NCT03124368			(103)
		NCT04609696	Phase I	Healthy	(104)
		NCT04551599			(105)
		NCT04709094			(106)
		NCT04451434			(107)
**ALXN2050** (ACH-5228/ACH-0145228)	Alexion Pharmaceuticals	NCT04170023	Phase II	PNH	(108)
		NCT04609670	Phase I	Healthy	(109)
		NCT04551586			(110)
		NCT04709081			(111)
		NCT04660890			(112)
		NCT04623710		Healthy with kidney impairment	(113)
**BCX9930**	BioCryst Pharmaceuticals	NCT04702568	Phase II	PNH	(114)
		NCT04330534	Phase I	Healthy and PNH	(115)

C3G, complement 3 glomerulopathy; C3GN, C3 glomerulonephritis; DDD, dense deposit disease; IC-MPGN, immune complex membranoproliferative glomerulonephritis; PNH, paroxysmal nocturnal hemoglobinuria.

One inhibitor is ALXN2040, which, after showing positive results in preclinical studies for the treatment of PNH and aHUS (89), entered Phase I, II, and III studies. In a dose-finding, open-label Phase II study (NCT03053102), ALXN2040 as monotherapy was found to inhibit intravascular hemolysis and improve hemoglobin levels in previously untreated patients with PNH (116). Patients in this study were given 100–200 mg of the drug three times a day orally, and it was found to be well tolerated. Despite these promising results, however, it was noted that residual alternative pathway activity was observed in some of the patients. In another Phase II study (NCT03472885), PNH patients with inadequate response to eculizumab were given 100–200 mg ALXN2040 three times daily, in addition to their eculizumab regimen, over 24 weeks (117). In this study, ALXN2040 resulted in an increase in hemoglobin levels at Week 24 (mean increase, 2.4 g/dL) and reduced transfusion requirements compared to the pre-ALXN2040 treatment period. Currently, a Phase III study (NCT04469465) examining the therapeutic value of ALXN2040 as an add-on therapy to a C5 inhibitor is underway in patients with PNH who develop extravascular hemolysis (98).

The other small-molecule factor D inhibitors that have progressed into clinical trials are ALXN2050 and BCX9930, both of which are currently being investigated in Phase II studies for long-term safety and efficacy in the treatment of PNH (108, 114). Congress abstracts of a Phase I study of BCX9930 (NCT04330534) demonstrated that BCX9930 as oral monotherapy can achieve complete and durable suppression of the alternative pathway and elicit rapid clinical changes that are indicative of reduced hemolysis (118, 119). BCX9930 was also well tolerated over a range of doses (10–1,200 mg for single-dose evaluations and 50–400 mg twice a day for multiple-dose evaluations).

In contrast to small-molecule inhibitors, only one anti–factor D antibody has been evaluated in clinical trials for the treatment of alternative pathway–mediated diseases. This antibody, called lampalizumab (FCFD4514S; Genentech/Roche), is the antigen-binding fragment of a humanized monoclonal antibody directed against complement factor D (4, 120). Originally, it showed promising results in a Phase II clinical trial when administered by intravitreal injection to patients with GA secondary to age-related macular degeneration (120), but it did not reduce GA enlargement in two Phase III randomized clinical trials (121). Future studies with new antibody therapy strategies are warranted.

## Concluding Remarks

Like many biological processes, the alternative pathway and the wider complement system can function as a double-edged sword. Together, they protect the body against pathogen invasion and maintain a healthy state when properly controlled, but if dysregulated, they can lead to a wide range of detrimental effects. Although many of the diseases driven by complement dysregulation present local manifestations, systemic complement dysregulation is also common and, as such, both local inhibition and systemic inhibition may be required for disease control (90). Interestingly, although complement dysregulation is common to many diseases, very subtle differences result in extremely diverse disease phenotypes.

As factor D is an essential enzyme of the alternative pathway with concentrations lower than those of other complement proteins, it is the rate-limiting enzyme of this pathway. Accordingly, it is an important strategic target in diseases where the alternative pathway plays a central role (17). Furthermore, because factor D has a function in both physiological and disease processes, its therapeutic inhibition could offer a variety of clinical possibilities that go well beyond the treatment of the diseases discussed in this review. For example, although further work is needed to elucidate the role of factor D in intracellular signaling when secreted from senescent cells, perhaps its inhibition could help to control the negative influence of senescent cells in aging-related disorders, including disorders of the dermis. Furthermore, patients with chronic kidney disease have been found to have high levels of complement components, including increased levels of factor D in plasma microparticles (tiny membrane vesicles that are shed from cells in response to injury) (46). Microparticle-associated factor D has been linked to systemic activation of the alternative pathway and endothelial dysfunction. Moreover, as mentioned previously, complement-mediated diseases such as aHUS can also lead to kidney failure, which in turn can cause elevated levels of factor D because of poor glomerular filtration (24). Inhibiting factor D in all of these scenarios may help to prevent amplification of the alternative pathway and thereby limit systemic inflammation, organ damage, and disease progression.

Because the amplification loop of the alternative pathway also enhances the signal of the other two complement pathways, factor D inhibitors may also have a place in treating diseases that are initially triggered by those pathways. For example, neuromyelitis optica is an autoimmune disease of the central nervous system that is characterized by inflammatory demyelinating lesions in the spinal cord and optic nerve (122). These lesions can eventually lead to paralysis and blindness. Neuromyelitis optica is caused by an immunoglobulin G autoantibody (AQP4-IgG) that activates the classical complement pathway in the central nervous system (123). Elevated levels of terminal components of the complement cascade, including C5a and the MAC, have been found in the plasma and cerebrospinal fluid of patients with this disease and are linked with disease activity (124). As factor D and the alternative pathway play an important role in amplifying the effects of the classical pathway *via* the C3 amplification loop, they likely play a considerable role in the generation and deposition of these complement fragments. Therefore, it would be interesting to see whether factor D inhibitors could help to limit the detrimental effects of this disease.

Factor D inhibitors may also emerge as a treatment option for new diseases or those where the alternative pathway was not previously known to play a role. One recent example of this is COVID-19. Some patients with COVID-19 present with clinical features that resemble those seen in patients with other complement-dysregulated diseases, including inflammation, thrombotic microangiopathy, and endothelial damage (125). In fact, deposits of terminal complement components have been found both in the lung microvasculature of patients with COVID-19 suffering from pneumonia and in the cutaneous microvasculature of skin lesions (126). It has also been shown *in vitro* that spike proteins of the COVID-19 coronavirus bind to heparan sulfate on cell surfaces and interfere with the ability of factor H to regulate the activity of the alternative pathway (125). Importantly, the small-molecule factor D inhibitor ACH-145951 (Achillion Pharmaceuticals) was able to block the resulting activation of the alternative pathway and prevent subsequent cell lysis.

Although findings like these add to the list of evidence supporting a role for factor D inhibitors in modulating the alternative pathway and inhibiting the progression of complement-mediated diseases, rigorous clinical studies will be necessary. Careful monitoring of the long-term safety of factor D inhibitors and evaluating the risk of adverse or off-target effects will be paramount, especially because complement deficiencies are known to be associated with recurrent infections and an increased risk of autoimmune disorders (127). Importantly, the use of factor D or other complement inhibitors should always be evaluated on an individual basis to ensure that any clinical benefit outweighs the risk of jeopardizing critical immunological defense mechanisms.

The multifaceted nature of the alternative pathway and the complement system itself, together with the complex role they play in both human health and disease, renders therapeutic treatment of complement-mediated diseases a challenge. Further studies aimed at resolving some of the unanswered questions will prove critical for determining optimal targets and levels of inhibition so that physiological immune function is not compromised.

## Author Contributions

JB and IW both contributed to the conception of this manuscript, development of the first draft, and revision of subsequent drafts, and also read and approved the final submitted version. All authors contributed to the article and approved the submitted version.

## Funding

The authors declare that funding from BioCryst Pharmaceuticals, Inc., Durham, NC, United States, was provided for medical writing support and editorial assistance. The funder was not involved in the design or writing of this manuscript, or in the decision of where to submit it for publication.

## Conflict of Interest

JB is a member of the Scientific Advisory Board to BioCryst Pharmaceuticals, Inc., Durham, NC, United States. IW is a member of the Scientific Advisory Board to BioCryst Pharmaceuticals, Inc., Durham, NC, United States and receives honoraria from Alexion Pharmaceuticals, Inc., Boston, MA, United States.

## Publisher’s Note

All claims expressed in this article are solely those of the authors and do not necessarily represent those of their affiliated organizations, or those of the publisher, the editors and the reviewers. Any product that may be evaluated in this article, or claim that may be made by its manufacturer, is not guaranteed or endorsed by the publisher.
